# Differences in the composition of the bacterial element of the urinary tract microbiome in patients undergoing dialysis and patients after kidney transplantation

**DOI:** 10.3389/fmicb.2023.1187625

**Published:** 2023-06-07

**Authors:** Marcelina M. Jaworska, Paulina Pecyna, Katarzyna Jaskiewicz, Małgorzata Rydzanicz, Malgorzata Kaluzna, Krzysztof Pawlaczyk, Rafal Ploski, Dorota M. Nowak-Malczewska, Justyna A. Karolak, Marzena Gajecka

**Affiliations:** ^1^Chair and Department of Genetics and Pharmaceutical Microbiology, Poznan University of Medical Sciences, Poznan, Poland; ^2^Institute of Human Genetics, Polish Academy of Sciences, Poznan, Poland; ^3^Department of Medical Genetics, Medical University of Warsaw, Warsaw, Poland; ^4^Chair and Department of Endocrinology, Metabolism and Internal Diseases, Poznan University of Medical Sciences, Poznan, Poland; ^5^Department of Nephrology, Transplantology and Internal Medicine, Poznan University of Medical Sciences, Poznań, Poland

**Keywords:** urobiome, urinary tract infection, dialysis, renal transplantation, urine culture

## Abstract

**Introduction:**

The development of molecular biology methods and their application in microbial research allowed the detection of many new pathogens that cause urinary tract infections (UTIs). Despite the advances of using new research techniques, the etiopathogenesis of UTIs, especially in patients undergoing dialysis and patients after kidney transplantation, is still not fully understood.

**Methods:**

This study aimed to characterize and compare the composition of the bacterial element of the urinary tract microbiome between the groups of patients undergoing dialysis (*n* = 50) and patients after kidney transplantation (*n* = 50), with positive or negative urine culture, compared to healthy individuals (*n* = 50).

**Results:**

Asymptomatic bacteriuria was observed in 30% of the urine cultures of patients undergoing dialysis and patients after kidney transplantation, with *Escherichia coli* as the most dominant microorganism (73%) detected with the use of classical microbiology techniques. However, differences in the bacterial composition of the urine samples between the evaluated patient groups were demonstrated using the amplicon sequencing. *Finegoldia*, *Leptotrichia*, and *Corynebacterium* were found to be discriminative bacteria genera in patients after dialysis and kidney transplantation compared to the control group. In addition, in all of urine samples, including those without bacteriuria in classical urine culture, many types of bacteria have been identified using 16S rRNA sequencing.

**Discussion:**

The revealed microbial characteristics may form the basis in searching for new diagnostic markers in treatment of patients undergoing dialysis and patients after kidney transplantation.

## 1. Introduction

While historically urine has been considered sterile in healthy individuals, recent studies have revealed the presence of a wide variety of bacteria in the urinary tract that are usually unidentifiable by conventional microbiological techniques, but can be recognized using molecular methods, including the 16S amplicon sequencing (Wolfe et al., 2012; Lewis et al., 2013; Johnson et al., 2019).

Since the discovery of the human urobiome, which is defined as a group of microorganisms and their genomes occurring in the urinary system, some research has been done to characterize its composition and understand its relationship to human health and disease (Wolfe and Brubaker, 2019). Disturbances in the functioning of the bacterial element of the urinary tract microbiome can cause diseases of the urinary system such as overactive bladder, interstitial cystitis, or neurogenic bladder dysfunction (Pearce et al., 2014; Whiteside et al., 2015).

Urinary tract infection (UTI) is a serious problem in patients with chronic kidney disease (CKD), especially in individuals undergoing dialysis or kidney transplant (Grabe et al., 2015; Coussement et al., 2020). UTI is associated with increased risks of acute rejection, allograft dysfunction, graft loss, increased hospital stays, and mortality (Ariza-Heredia et al., 2014; Koga et al., 2022). Furthermore, a recurrent UTI, which occurs in 7% of the patients after kidney transplantation, is one of the leading causes of allograft loss and death (Britt et al., 2017). Therefore, prediction, early detection, and prevention of UTI are essential. In patients with CKD, UTI can be caused by both, opportunistic bacteria and uropathogens responsible for mixed infections (Naumnik and Myśliwiec, 2013). For many decades, knowledge about uropathogens was considered to be well-established and fairly consistent. Currently, the development of molecular biology methods and their application in research have allowed the identification of many new pathogens, also involved in the etiology of UTI (Price et al., 2018).

Knowledge about the bacterial component of the urinary tract in patients undergoing dialysis or in patients after kidney transplantation remains limited. Thus far, scarce information, including the differentiation of the bacterial element of the urinary tract microbiome depending on the gender of patients after kidney transplantation (Fricke et al., 2014) and differences in the microbiota composition between urine samples of patients after kidney transplantation and healthy individuals (Modena et al., 2017; Rani et al., 2017) have been reported. However, there is no data about differences in the urinary tract microbiome between patients undergoing dialysis and patients after kidney transplantation.

The detection of bacteria in urine samples using culturing methods is the main diagnostic criterion for UTI (Hryniewicz and Holecki, 2015). While culture-dependent techniques allow the identification of typical uropathogens, the isolation and identification of highly demanding and non-cultured microorganisms are not routinely performed in microbiological diagnostics of UTI Consequently, the role of highly demanding and non-cultured bacteria remains elusive in the etiology of UTIs (Magistro and Stief, 2019). Therefore, the molecular biology-based approach could be essential in the identification of bacteria in patients with negative urine cultures (Thomas-White et al., 2018; Wolfe and Brubaker, 2019).

Due to limited data on the bacterial component of the urinary tract microbiome in patients with kidney diseases, the aim of this research was to characterize and compare the composition of the bacterial element of the urinary tract microbiome between the groups of patients with positive or negative urine culture in classical microbiology undergoing dialysis or kidney transplant, compared to control individuals without UTI.

## 2. Materials and methods

### 2.1. Patient ascertainment and sample collection

Study individuals were recruited in the outpatient clinic and inpatient ward in the Chair and Department of Endocrinology, Metabolism, and Internal Diseases at the Poznan University of Medical Sciences (PUMS). Patients with end-stage-renal diseases (ESRD), including patients after renal transplantation and patients undergoing dialysis, as well as individuals without urinary tract dysfunction (controls), were enrolled in this study. Patients were assigned to the adequate study group based on the ESRD-treatment. The inclusion criterion for the control group was lack of active UTI. Antibiotic/antifungal treatment 3 weeks before the urine sample collection was the exclusion criterion in the subgroups of patients and controls.

A urine sample was obtained once from each study participant by a sterile mid-stream collection. Derived samples were divided into two aliquots—the first part was immediately used for the assessment by techniques of classical microbiology, while the second part was centrifuged at 800 rcf for 10 min and the pellet was stored at −80°C for the genetic testing.

The study was carried out in accordance with the research protocol approved by the Bioethics Committee at the PUMS (no. 942/14 (2014.12.04), 191/15 (2015.02.05) and 1170/19 (2019.12.05)). The possible consequences of the study were explained, and informed consent was obtained from all participants, according to the Declaration of Helsinki.

### 2.2. Assessment of bacterial cultures of urine samples

Urine samples were inoculated by a Hoeprich quantitative method on the chromogenic medium Chrom ID^®^ CPS Elite (Biomerieux), then used for isolation, quantification, and presumptive identification of *Enterococcus*, *Proteeae* (*Proteus, Providencia, Morganella*), KESC group (*Klebsiella, Enterobacter, Serratia, Citrobacter*), *Staphylococcus saprophyticus,* and *Streptococcus agalactiae*. After incubation at 35–37°C for 24–48 h, the grown colonies were counted and reported as the number of colony forming units (CFUs) per 1 mL of urine sample.

### 2.3. DNA isolation

Genomic microbial DNA was extracted from urine pellets using Genomic Mini AX Body Fluids extraction kit (A&A Biotechnology), according to the manufacturer’s protocol with one modification, in a form of adding 10 μL of each of mutanolysin (activity >10,000 U/mg), lysostaphin (concentration: 15 U/μL), lysozyme (concentration: 10 mg/mL), and lyticase (concentration: l0 U/μL) to ensure cell lysis.

### 2.4. 16S rRNA amplicon sequencing

All steps were performed according to the 16S Metagenomic Sequencing Library Preparation—Preparing 16S Ribosomal RNA Gene Amplicons for the Illumina MiSeq System protocol (Illumina). Briefly, V3 and V4 hypervariable regions of the bacterial 16S rRNA gene were amplified using 515F and 806R primers. Libraries were prepared with Nextera XT Index Kit (Illumina) followed by paired-end sequencing (2×300 bp) using MiSeq System (Illumina, San Diego, CA, United States). The sequencing run was performed with 10% of reference PhiX Control v3 Library (Illumina) spike-in to improve sequencing quality of 16S rRNA amplicon low diversity libraries. The sequencing was performed at the Medical University of Warsaw in Poland.

### 2.5. Bioinformatic analysis and taxonomic assignment

Bioinformatic analysis of obtained 16S rRNA amplicon sequence reads was performed by the bioinformatics company, ideas4biology Sp. z o.o. in Poznan, Poland, in accordance with the previously described protocol (Katoh et al., 2002; McDonald et al., 2012; Callahan et al., 2016; Bokulich et al., 2018; Bolyen et al., 2019). Analyzes of 16Sr RNA amplicon sequencing data were performed using QIIME 2 version 2019.7 (Bolyen et al., 2019). The control and improvement of the quality of the readings (including the removal of phiX and chimeras) was based on the q2-dada2 function implementing the DADA2 algorithm (Callahan et al., 2016). The obtained amplicon sequence variants (ASVs) were aligned using the mafft algorithm (Katoh et al., 2002) implemented as part of the q2-alignment function. ASVs are generated by a *de novo* process, in which biological sequences are distinguished on the basis of more frequent repetition than sequence error, and are applied to individual DNA sequences recovered from high-throughput sequencing. The taxonomic affiliation was assigned to the ASV with the help of a naive Bayesian class fitter trained using the q2-feature-class plugin (Bokulich et al., 2018). The Greengenes 13_8 99% OTUs reference sequences database (McDonald et al., 2012) was used to teach the fi cator class, taken from the ftp address: http://qiime.org/home_static/dataFiles.html Alpha-diversity (Shannon’s diversity index) and beta-diversity (Bray-Curtis distance) analyzes were carried out. The overall structural similarity and variation (beta diversity between samples) between the bacterial element of urine microbiomes from the patients undergoing dialysis, patients after kidney transplantation, and controls were then examined using the Bray–Curtis dissimilarity distance analysis with Principal Coordinates Analysis (PCoA).

### 2.6. Statistical analyzes

Statistical analyzes were performed using Statistica, version 13.4 (Dell Inc.). The compliance of the empirical data distributions with the normal distribution was verified with the Shapiro–Wilk W test. Due to the fact that the distribution of data was not consistent with the normal distribution, the analysis of differences between the study groups was assessed using the Kruskal-Wallis test with the post-hoc multiple comparison test (Dunn’s test).

### 2.7. Comparison of the bacterial element of the urinary tract microbiome between patients undergoing dialysis and patients after kidney transplantation

In order to differentiate bacterial species identified in the 16S rRNA amplicon sequencing between the samples in the groups of patients undergoing dialysis and patients after kidney transplantation, with the positive or negative results of urine culture, statistical analyzes were performed using the chi square test. The analyzes were carried out in the following scheme: (i) patients undergoing dialysis with a positive urine culture vs. patients undergoing dialysis with a negative urine culture, (ii) patients after kidney transplantation with a positive urine culture vs. patients after kidney transplantation with a negative urine culture, and (iii) patients undergoing dialysis and patients after kidney transplantation with a positive urine culture vs. patients undergoing dialysis and patients after kidney transplantation with a negative urine culture.

## 3. Results

### 3.1. Characteristics of patients and controls

In total, 100 patients and 50 control individuals, with the age range of 18–80 years participated in the study. In the group of patients undergoing dialysis, 15 and 35 individuals were treated with peritoneal dialysis (DO) or hemodialysis (HD), respectively. In Table 1 demographic characteristics of study participants are compiled.

**Table 1:** Demographic characteristics of the studied groups.

Age ranges (years)	Patients undergoing dialysis (*n* = 50)		Patients after kidney transplantation (*n* = 50)		Controls (*n* = 50)	
	Female	Male	Female	Male	Female	Male
<30	1 (2%)	1 (2%)	1 (2%)	0 (0%)	9 (18%)	0 (0%)
31–40	1 (2%)	5 (10%)	6 (12%)	10 (20%)	10 (20%)	0 (0%)
41–50	3 (6%)	3 (6%)	2 (4%)	2 (4%)	2 (4%)	3 (6%)
51–60	4 (8%)	7 (14%)	4 (8%)	11 (22%)	7 (14%)	3 (6%)
61–70	12 (24%)	6 (12%)	7 (14%)	5 (10%)	9 (18%)	3 (6%)
>70	2 (4%)	5 (10%)	0 (0%)	2 (4%)	3 (6%)	1 (2%)
Total	23 (46%)	27 (54%)	20 (40%)	30 (60%)	40 (80%)	10 (20%)

Age ranges and gender of study participants in the groups of patients undergoing dialysis, patients after kidney transplantation, and controls.

Polycystic kidney disease and glomerulonephritis were the most common indications for treatment with DO and HD, respectively. In 6% of patients, the cause of the renal dysfunction leading to the decision to apply dialysis therapy was undetermined nephropathy.

Glomerulonephritis was the most common indication for kidney transplantation. In 22% patients, the cause of the renal dysfunction remained undetermined nephropathy. Diseases identified in patients undergoing dialysis or patients after kidney transplantation are listed in Table 2.

**Table 2:** Primary disease or urinary system dysfunction, treated with dialysis or resulting in a kidney transplant in studied patients.

Primary disease/comorbidities	Classification of causes of CKD*	Studied groups		
		Patients undergoing dialysis (*n* = 50)	Patients after kidney transplantation (*n* = 50)	Total (*n* = 100)
Hypertension (HT)	Vascular diseases	8 (16%)	8 (16%)	16 (16%)
HT and urinary system defects	Vascular diseases and cystic and congenital diseases	0 (0%)	1 (2%)	1 (1%)
HT and proteinuria	Vascular diseases	0 (0%)	1 (2%)	1 (1%)
Glomerulonephritis (GN)	Glomerular diseases	10 (20%)	11 (22%)	21 (21%)
GN and HT	Glomerular diseases and vascular diseases	2 (4%)	1 (2%)	3 (3%)
Obstructive nephropathy	Tubulointerstitial diseases	0 (0%)	1 (2%)	1 (1%)
Reflux nephropathy	Cystic and congenital diseases	0 (0%)	1 (2%)	1 (1%)
Chronic tubulointerstitial nephropathy	Tubulointerstitial diseases	0 (0%)	1 (2%)	1 (1%)
Chronic glomerulonephritis (cGN)	Glomerular diseases	0 (0%)	2 (4%)	2 (2%)
(cGN) and HT	Glomerular diseases and vascular diseases	0 (0%)	1 (2%)	1 (1%)
Polycystic kidney disease	Cystic and congenital diseases	9 (18%)	2 (4%)	11 (11%)
Diffuse mesangial proliferative nephritis	Glomerular diseases	0 (0%)	2 (4%)	2 (2%)
Diabetes	Glomerular diseases	0 (0%)	5 (10%)	5 (5%)
Diabetes and HT	Glomerular diseases and vascular diseases	0 (0%)	2 (4%)	2 (2%)
GN and cirrhosis of the right kidney	Glomerular diseases	1 (2%)	0 (0%)	1 (1%)
Tubulointerstitial nephropathy (TN)	Tubulointerstitial diseases	6 (12%)	0 (0%)	6 (6%)
TN and urinary system defects	Tubulointerstitial diseases and cystic and congenital diseases	2 (4%)	0 (0%)	2 (2%)
Chronic renal failure (CHF)—exact cause unknown	Unknown nephropathies	5 (10%)	0 (0%)	5 (5%)
Hypertensive nephropathy	Vascular diseases	2 (4%)	0 (0%)	2 (2%)
Wegener’s granuloma	Glomerular diseases	2 (4%)	0 (0%)	2 (2%)
Cause unknown	Unknown nephropathies	3 (6%)	11 (22%)	3 (3%)
Total		50 (100%)	50 (100%)	100 (100%)

*Classification of causes of CKD according with National Kidney Foundation (https://www.kidney.org/professionals/explore-your-knowledge/how-to-classify-ckd).

### 3.2. Characteristics of microorganisms identified in urine cultures

The etiological factor responsible for UTI was identified (bacteriuria ≥10^5^ CFU/mL) in 30% of the samples obtained from patients undergoing dialysis and patients after kidney transplantation. Asymptomatic bacteriuria was found to be more common in women (18/43 = 42%) than in men (13/57 = 23%). In the group of patients undergoing dialysis, asymptomatic bacteriuria was recognized in 8/23 women (35%) and 7/27 men (26%), while in patients after kidney transplantation, asymptomatic bacteriuria was recognized in 10/20 women (50%) and 5/30 men (17%) *Escherichia coli* was the most common etiological factor of asymptomatic bacteriuria and was detected in 80% of patients undergoing dialysis and 67% of patients after kidney transplantation. *Staphylococcus* spp., *Enterococcus* spp., *Kocuria* spp., *Morganella morganii*, and *Enterobacter cloacae complex* were also recognized as etiological factors of asymptomatic bacteriuria in these study groups. Detailed information about bacteria identified in urine cultures is presented in Supplementary Tables S1–S3.

### 3.3. The composition of the bacterial element of urinary tract microbiome varies between patients undergoing dialysis and patients after renal transplantation

The bacterial DNA was extracted from all of the collected 150 urine samples. Out of them, 148 samples met the required qualitative and quantitative criteria and were subjected to the 16S rRNA amplicon sequencing.

Without taking into account the division into study groups, *Actinobacteria* (100%), *Firmicutes* (97%), *Proteobacteria* (91%), *Fusobacteria* (50%), *Cyanobacteria* (40%), *Acidobacteria* (22%), *Bacteroidetes* (16%), and *Thermi* (4%) were found as the most common types of bacteria detected in the assessed samples. The substantial difference in bacteria abundance in urine samples was detected for *Proteobacteria* between patients undergoing dialysis and controls (*p* = 0.047), and between patients undergoing dialysis and patients after renal transplantation (*p* = 0.037). The superior abundance was found in a group of patients undergoing dialysis.

**Figure 1:**
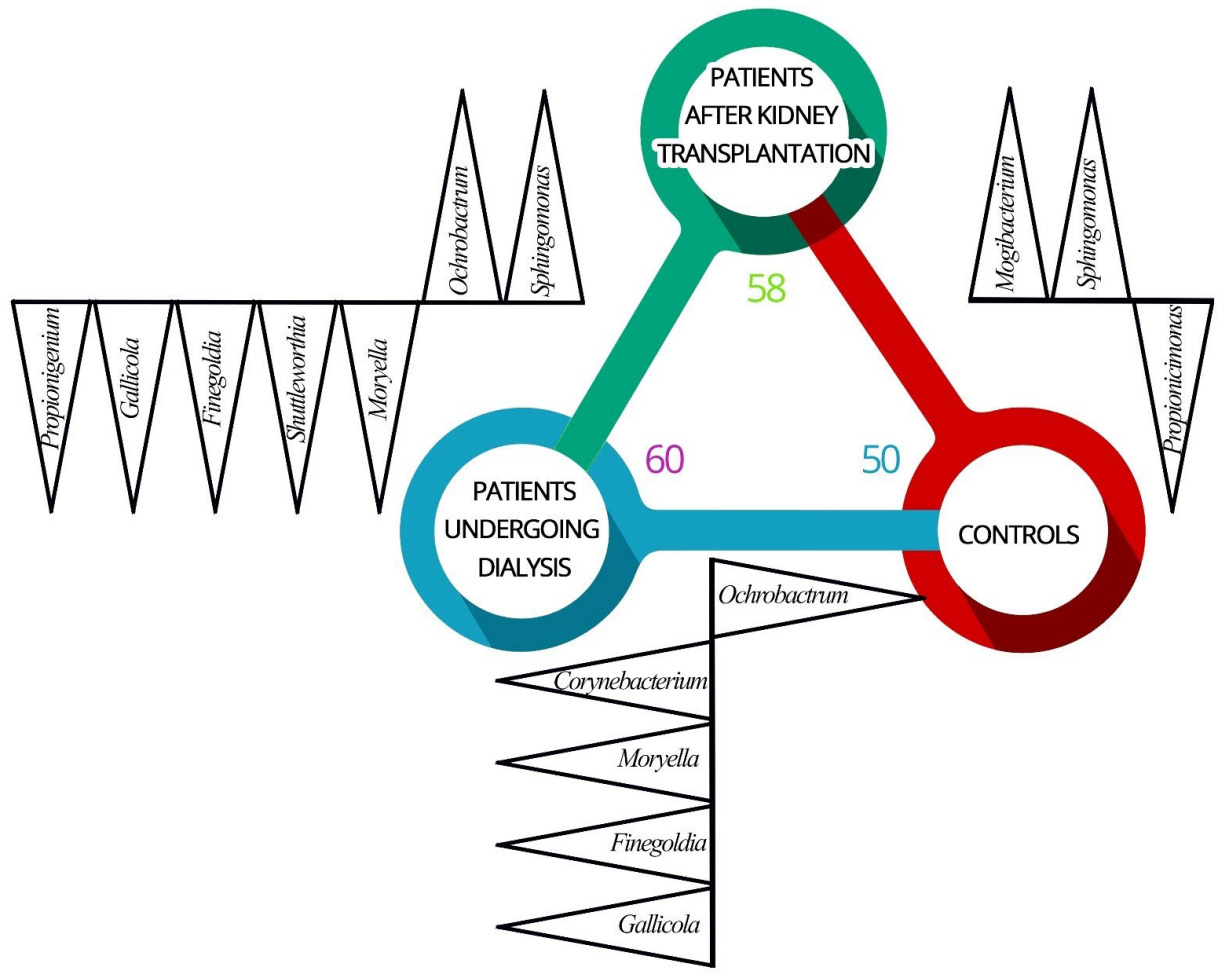
Differences in the composition of the bacterial element of urinary tract microbiome between patients undergoing dialysis (D), patients after renal transplantation (T), and control individuals (C). Identified genera (black triangles) of the bacterial element of the urinary tract microbiome varies between patients undergoing dialysis (blue circle), patients after kidney transplantation (green circle), and control individuals (red circle). Increased abundance in (D) vs. (C) were found for *Corynebacterium*, *Moryella*, *Finegoldia,* and *Gallicola. Ochrobactrum* was identified more often in (C) comparing to (D). *Mogibacterium* and *Sphingomonas* were more often identified in (T) comparing to (C). *Propionicimonas* was identified with a higher abundance in (C) compared to (T). Increased abundance of genera *Moryella*, *Shuttleworthia*, *Finegoldia*, *Gallicola*, and *Propionigenium* was recognized in (D) and *Ochrobactrum* and *Sphingomonas* were detected with higher abundance in (T) compared to (D).

Overall, at the genus level, 76 different genera were detected in the samples derived from patients. The highest number of genera were identified in patients undergoing dialysis (*n* = 60), followed by patients after renal transplantation (*n* = 58). In urine samples obtained from controls, 50 different genera were detected. The number and variability of identified bacteria genera of the bacterial element of the urinary tract microbiome between groups are presented in Figure 1.

The genera *Corynebacterium* (*p* = 0.002), *Moryella* (*p* = 0.033), *Finegoldia* (*p* < 0.001), and *Gallicola* (*p* = 0.002) were detected with increased abundance in urine samples of patients undergoing dialysis comparing to urine samples derived from controls. Representatives of the genus *Ochrobactrum* were identified more often in controls compared to patients undergoing dialysis (*p* < 0.0001).

Bacteria from genera *Mogibacterium* (*p* = 0.049) and *Sphingomonas* (*p* < 0.0001) were more often identified in the urine samples from patients after renal transplantation compared to controls. The sequences corresponding to the genus *Propionicimonas* were identified with a higher abundance in urine samples derived from controls (*p* = 0.041).

Differences in relative abundances of selected genera were also observed between urine samples obtained from patients undergoing dialysis and patients after kidney transplantation. Increased abundance of genera *Moryella* (*p* = 0.033), *Shuttleworthia* (*p* = 0.003), *Finegoldia* (*p* < 0.001), *Gallicola* (*p* = 0.002), and *Propionigenium* (*p* = 0.001) was recognized in patients undergoing dialysis. On the other hand, the genera *Ochrobactrum* (*p* < 0.0001) and *Sphingomonas* (*p* < 0.0001) were detected with higher abundance in urine samples from renal transplant patients.

**Figure 2:**
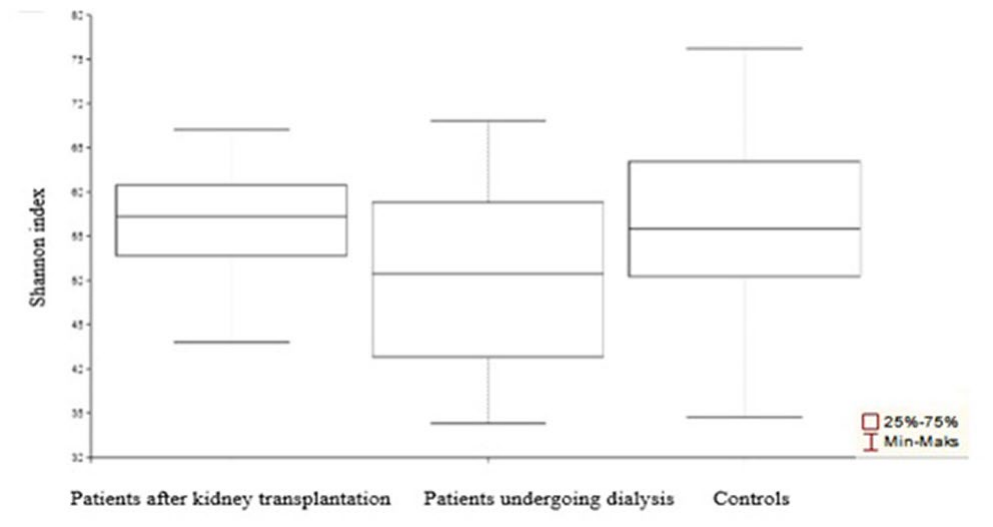
α-Diversity of the urinary tract microbiome bacterial element among patients after transplantation, undergoing dialysis, and control individuals. Compared to control samples, the most diverse was the urine microbiota composition among patients undergoing dialysis.

The obtained values of alpha-diversity indicate heterogeneity of the samples (Figure 2). Bacterial relative abundance in samples derived from patients undergoing dialysis and patients after kidney transplantation was found to be larger as compared to control samples (*p* = 0.003 and *p* = 0.004, respectively). There was no significant difference in the relative abundance of bacteria in patients after renal transplantation compared to patients undergoing dialysis (*p* = 0.68).

**Figure 3:**
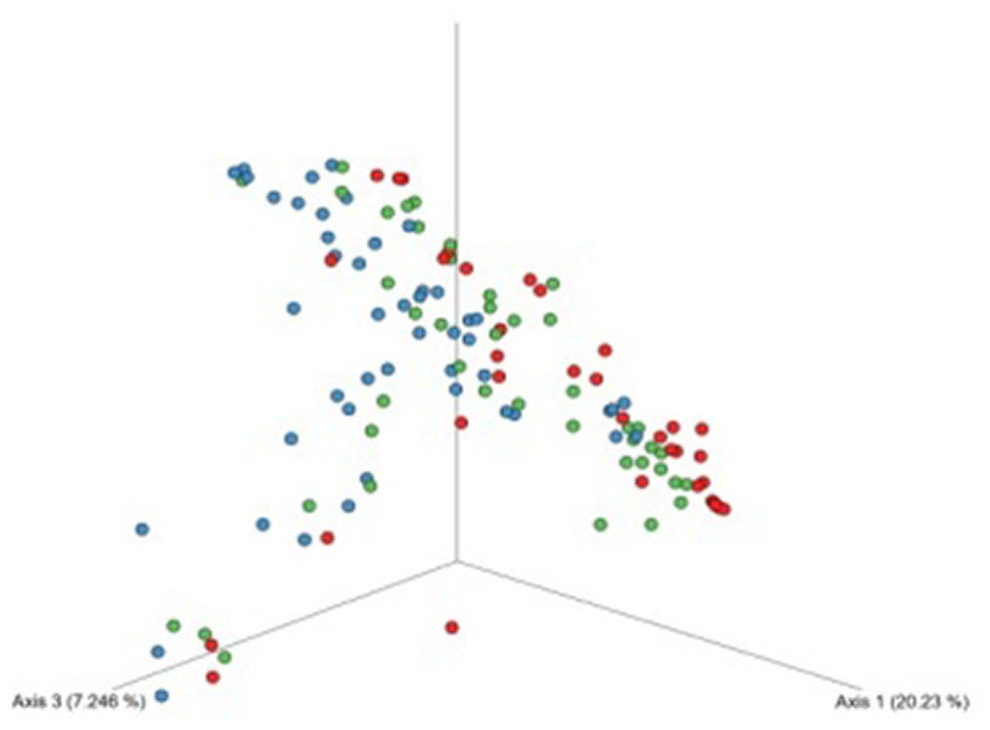
Beta-diversity assessed by the Bray–Curtis dissimilarity tests between patients undergoing dialysis (blue dots), patients after kidney transplantation (green dots), and controls (red dots) based on ASVs. The bacterial element of urine microbiota of patients undergoing dialysis was more variable in composition of microorganisms.

The analyses of bacterial structural similarity and variation revealed substantial differences between samples were detected and the results are presented in Figure 3. Bacterial element of the urinary tract microbiome in samples from patients undergoing dialysis was the most diverse in terms of composition. On the other hand, urine samples from controls were characterized by the smallest diversity.

### 3.4. Composition of the urinary tract microbiome in patients with positive vs. negative urine cultures

**Figure 4:**
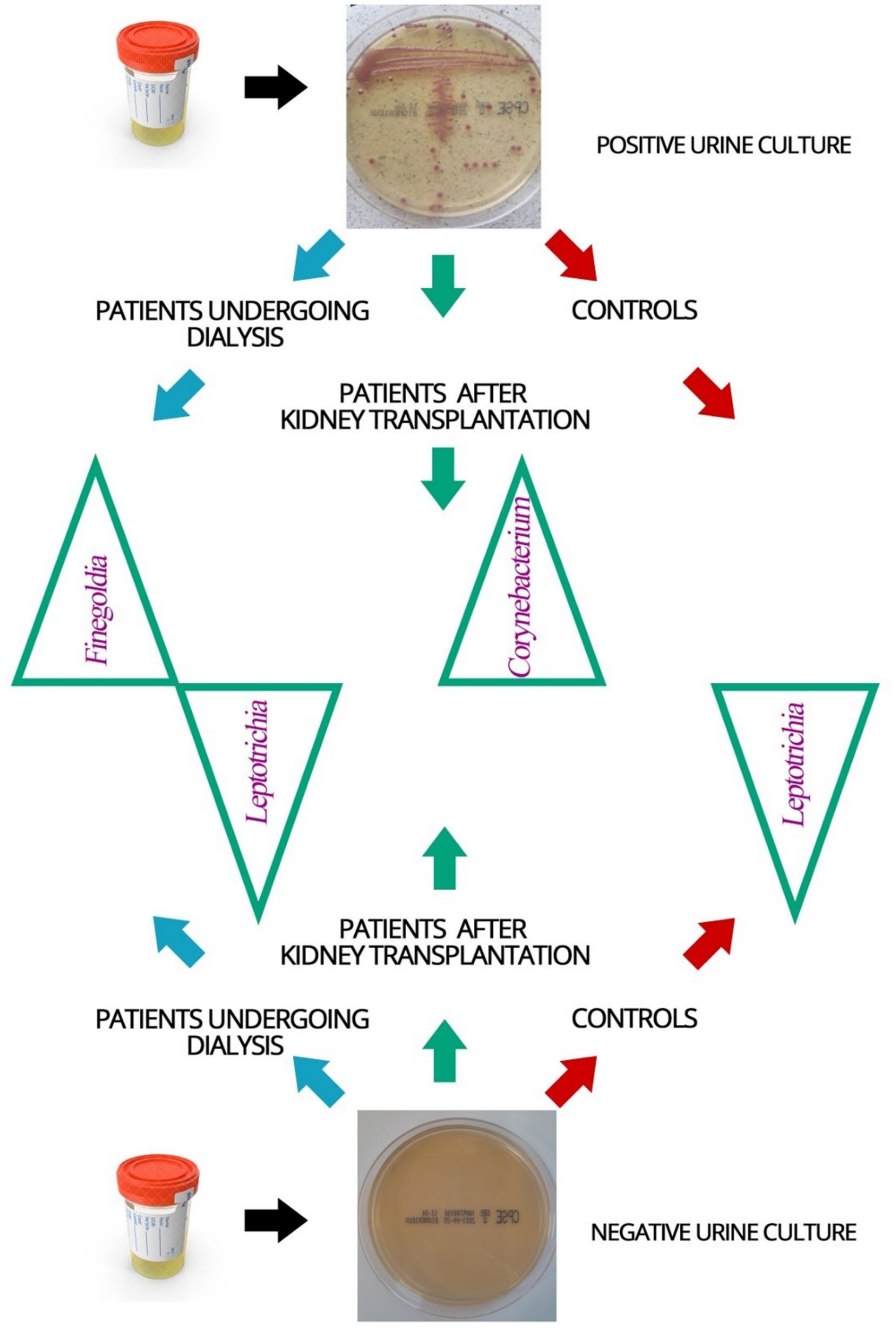
Schematic representation of the bacterial element of the urinary tract microbiome identified in the urine samples of patients and control individuals with positive and negative urine cultures. Blue arrow—dialysis patients, green arrow—patients after kidney transplantation, red arrow—controls; green triangles—names of identified bacteria genera of the bacterial element of the urinary tract microbiome that were found to be discriminative in the studied groups of patients.

Bacterial element of the urinary tract microbiome in patients with positive and negative urine cultures was compared (Figure 4). In urine samples derived from patients with positive urine culture in classical microbiology, lower diversity of bacterial genera comparing to samples from patients with negative urine culture was found in the 16S amplicon sequencing. In addition, bacteria from *Finegoldia*, *Leptotrichia*, and *Corynebacterium* genera were found to be discriminative in patients undergoing dialysis and patients after kidney transplantation compared to individuals without urinary tract dysfunction.

In urine samples from patients undergoing dialysis, with a negative urine culture, bacteria *Leptotrichia* were identified more frequently (*p* = 0.003), and those from the genus *Finegoldia* less often (*p* = 0.038) than in the group of patients undergoing dialysis with a positive urine culture.

*Corynebacterium* bacteria were recognized less often in the urine samples of kidney transplant patients with negative urine culture than in positive urine culture samples from this group (*p* = 0.016).

In patients undergoing dialysis and kidney transplant patients with a negative urine culture, *Leptotrichia* was identified in the larger number of samples (*p* = 0.037) than in patients undergoing dialysis and kidney transplant patients with a culture-positive urine.

These results point to *Leptotrichia, Finegoldia* and *Corynebacterium* as potential diagnostics indicators of UTI.

## 4. Discussion

Chronic renal failure is a global health problem (Qiu et al., 2017) with the overall incidence of 242 people per million and an annual increase of 8% (Kim, 2019). In Poland, about 4.2 million people suffer from CKD (Gellert et al., 2020). Since the prevalence of CKD is calculated based on the number of patients with the end-stage disease only (Włodarczyk and Felcenloben, 2017–2018), the actual prevalence of CKD could be underestimated, both in Poland and worldwide (Włodarczyk and Felcenloben, 2017–2018).

UTI complication in patients undergoing dialysis is a significant problem in the process of renal replacement therapy that increases the frequency of hospitalization and the risk of death (Gołębiewska et al., 2011) Also, in the group of kidney recipients, UTI is a leading cause of post-transplant infectious complications in both men and women (Abbott et al., 2004; Tenke et al., 2008; Naber et al., 2010). In this study, asymptomatic bacteriuria, defined in accordance with the current recommendations (Hryniewicz and Holecki, 2015), was detected in 30% of patients undergoing dialysis or kidney transplantation.

Typical pathogens of the urinary tract are also most often responsible for asymptomatic bacteriuria in patients undergoing dialysis and patients after kidney transplantation. Thus, the results of our study are in line with previous reports showing the presence of *Escherichia coli* in 70% (Willner et al., 2014), 72.9% (Michno et al., 2018), or 82% (Olenski et al., 2019) of the urine samples with a positive culture result.

In order to extend the knowledge about urobiome, we evaluated groups of patients rarely included in microbiome studies, namely patients undergoing dialysis and patients after kidney transplantation. Assessment of the bacterial composition using 16S amplicon sequencing revealed 60 and 58 genera in urine samples from patients undergoing dialysis and patients after kidney transplantation, respectively. In controls, 50 different genera were recognized.

Culture-based methods, compared to the 16S rRNA amplicon sequencing, are less effective in detecting bacteria in urine samples and identify a limited number of microorganisms, mainly aerobic and fast-growing bacteria such as *Escherichia coli* (García and Isenberg, 2010; Wolfe and Brubaker, 2015). Using classical microbiology techniques, four genera of microorganisms were detected in urine samples from individuals without urinary tract dysfunction, 12 genera of bacteria in urine samples from patients undergoing dialysis, and 16 genera of microbes in urine samples from patients after kidney transplantation. In contrast, using 16S rRNA sequencing, we recognized higher bacterial heterogeneity, 58 genera were recorded in urine samples from patients after kidney transplantation and 60 genera of bacteria in the group of patients undergoing dialysis. Also, 37 detected genera were found to be common to all study groups.

Since as many as 80% of the phylotypes detected by 16S rRNA sequencing correspond to bacterial species that cannot be cultured *in vitro* (Jach and Sajnaga, 2017), the differences in the number of recognized microbes identified in our study are coherent. Such large disproportion in the number of bacterial genera obtained due to classical microbiology and 16S rRNA sequencing, as well as the identification of non-cultivated microorganisms, gave the opportunity to get a more complete picture of the microbiome and highlighted the role of molecular biology methods in the clinical examination of urine samples. Therefore, a combination of 16S rRNA gene sequencing along with quantitative urine culture would provide a reliable characterization of microbial communities in the urinary tract, since culture-based approaches would allow an approximate bacterial quantification and would therefore be useful as clinically relevant indicators (Perez-Carrasco et al., 2021).

Bacteria of the genera *Corynebacterium*, *Moryella*, *Finegoldia*, and *Gallicola* were more often identified in urine samples from patients undergoing dialysis than in control individuals, in which the dominant genus was *Ochrobactrum*. It has been reported that the midstream urinary microbiomes varied in adult patients with CKD and that variability was lower in more advanced CKD (Kramer et al., 2018). In recent years, there has been an interest in rare or infrequently described *Corynebacterium* species, which are emerging opportunistic pathogens considered to be potential uropathogens due to their detection in patients with UTIs (Barberis et al., 2021). *Corynebacterium phoceense* has been isolated from the urine of a kidney transplant recipient (Cresci et al., 2016). *Ochrobactrum anthropi* is considered atypical for dialysis peritonitis, a major complication of peritoneal dialysis therapy, as well as for other human infections (Gałgowska et al., 2020). However, this bacterium is an increasingly recognized cause of infection in immunocompromised hosts such as patients with kidney failure treated by dialysis (Abbassi-Nik et al., 2022).

Also, the composition of the urobiome in patients after kidney transplantation has not been sufficiently evaluated. Rani et al. indicated that urine microbiota in kidney transplant recipients was less diverse and dominated by potentially pathogenic Gram-negative bacteria of the genus *Escherichia* or *Enterobacter*, while urine microbiome in controls showed greater diversity and a higher incidence of non-pathogenic Gram-positive organisms such as *Propionibacterium*, *Corynebacterium*, and *Mobiluncus* (Rani et al., 2017). The study showed significant differences in the presence of bacteria of the genera *Mogibacterium* and *Sphingomonas*, which were more often identified in urine samples from transplant patients than in controls (Rani et al., 2017). The results of these studies may suggest that under the multiple stressors associated with kidney transplantation (including antibiotics or immunosuppression), the urinary microbiota of kidney-transplant recipients can decrease in diversity compared to healthy controls, while the abundance of opportunistic pathogens may increase, which may favor the promotion of antibiotic resistance (Rani et al., 2017).

*Mogibacterium* are Gram-positive, strictly anaerobic bacilli present in the oral environment and involved in oral infections (Casarin et al., 2012). Previously, the role of these microorganisms as the cause of urolithiasis was evaluated and the study concluded that differences and interactions between gut bacteria might predict certain types of urolithiasis (Zhao et al., 2020). The genus *Sphingomonas* is comprised of more than 30 species including human pathogen, *Sphingomonas paucimobilis* (Ryan and Adley, 2010). The most *Sphingomonas* infections are nosocomial and usually occur in immunocompromised individuals, including the kidney transplant patients (Ryan and Adley, 2010; Yu et al., 2015).

In a pilot study on the urinary microbiome of 21 renal transplant recipients and nine patients with acute kidney injury, rich and differentiated microbiome was found (Gerges-Knafl et al., 2020). Also, the same study demonstrated significant differences in the certain bacterial genera profiles between renal transplant recipients and patients with acute kidney injury, but the overall diversity did not differ between the two groups (Gerges-Knafl et al., 2020). In our study, comparing the urinary microbiota of patients undergoing dialysis and patients after transplantation, genera of *Sphingomonas*, *Ochrobactrum*, and *Actinocorallia* were found with a higher frequency in patients after kidney transplantation. On the other hand, genera *Moryella*, *Shuttleworthia*, *Finegoldia*, *Gallicola,* and *Propionigenium* were identified in urine samples from patients undergoing dialysis with a significantly higher frequency than in urine samples from patients after kidney transplantation. In the future research, numerous and longitudinal sampling of the patient’ urinary microbiome should be implemented to detect deviations from microbiome stability. If these changes indicate a risk of organ damage or loss, analysis of microbiome could be useful as a non-invasive method for early detection of transplanted organ failure (Campbell et al., 2020).

The genus *Ochrobactrum* includes nine species, with *Ochrobactrum anthropi*, Gram-negative oxygen-rod, increasingly recognized as a potentially problematic, opportunistic, and nosocomial pathogen (Hagiya et al., 2013). Asymptomatic bacteriuria associated with the administration of anti-thymocytic globulin contaminated with *Ochrobactrum anthropi* have been described in organ recipients (Gałgowska et al., 2020). Other complications caused by *Ochrobactrum anthropi* were associated with a superinfection of tissues implanted from deceased donors or contamination of the preservative fluid (Gałgowska et al., 2020).

*Moryella indoligenes* is a strictly anaerobic bacterium from the *Lachnospiraceae* family of the order *Clostridiales* (Carlier et al., 2007). Bacteria belonging to the genus *Moryella* were also detected in urine samples (Sabat et al., 2017) and the first case of bacteriemia involving this microorganism has been described in a patient with a history of prostatic hyperplasia complicated by recurrent UTI (Hansen et al., 2020).

*Shuttleworthia* is another discriminative type of bacteria found in our study. Previously *Shuttleworthia* has been suggested as a potential biomarker of inflammation in the urinary tract (Ling et al., 2017).

Here, the genus *Finegoldia* (formerly *Peptostreptococcus*) was identified in a high percentage in all three study groups with the highest number detected in the group of patients undergoing dialysis. *Finegoldia* are Gram-negative anaerobic opportunistic microorganisms that colonize the skin and mucous membranes (Song and Finegold, 2007; Murphy and Frick, 2013). The representative of this genus, *Peptostreptococcus prevotii* was often identified in men with non-gonococcal urethritis, while *Peptostreptococcus magnus* was the most common anaerobic species detected in patients without urethritis (Tungland, 2018). *Finegoldia* is also present in the urine of both sexually active adult males and sexually inactive adolescents (Tungland, 2018). In another study, two specific species of *Finegoldia* found in the urine of the bladder, including *Finegoldia magna*, were associated with preoperative worsening of urinary symptoms in women undergoing stress urinary incontinence and pelvic prolapse (Fok et al., 2018).

A comparison of 16S rRNA amplicon sequencing results in mid-stream urine samples from renal transplant patients with confirmed interstitial fibrosis and tubular atrophy (IFTA) and healthy controls showed that in IFTA patients, *Streptococcus* and *Lactobacillus* were more commonly identified than in controls (Modena et al., 2017). In patients with IFTA an increase in the number of pathogenic bacteria, *Propionibacterium acnes* (formerly *Cutibacterium acnes*), *Prevotella disiens*, *Gardnerella vaginalis*, and *Finegoldia magna* was found (Modena et al., 2017). It has been suggested that as the enrichment of uropathogens increases, the immune response in IFTA patients may be enhanced. In turn, it might result in an increased risk of transplant rejection in these individuals (Modena et al., 2017).

In another study, Colas et al. identified major changes in the urinary microbiota of patients following kidney transplantation with a significant impact on recipient status (spontaneously tolerant patient—TOL; minimal immunosuppressed patient—MIS; stable patient—STA). Their results highlighted a unique and specific urinary microbiota associated with spontaneous tolerance characterized by a high diversity and a clear *Proteobacteria* profile. In addition, TOL recipients were characterized by a specific and unique increase in *Proteobacteria* phylum (*Oxalobacteraceae* [genus *Janthinobacterium*]*, Caulobacteraceae, Comamonadaceae, Moraxellaceae* [*genus Acinetobacter*], *Xanthomonadaceae, Achromobacter, Yersinia*) (Colas et al., 2020).

In another study performed to identify differences in the urinary microbiome associated with chronic allograft dysfunction (CAD), *Corynebacterium* was more prevalent in female and male patients with CAD compared to non-CAD female patients. On the other hand, analysis of male CAD and female CAD patients showed greater abundance of phylum *Proteobacteria* in males (Wu et al., 2018).

In our study, 30% of patients undergoing dialysis and also 30% of patients after kidney transplantation had a positive urine culture with identification of an etiological factor indicative of UTI in classical microbiology. The impact of UTI on distant graft function is not fully understood. The predominance of pathogenic Gram-negative bacteria in urobiome of patients after kidney transplantation, indicated in some reports, highlights the problem of UTIs in recipients (Sabé et al., 2022) and a negative impact of early or late UTIs on the functioning of the transplanted kidney has been confirmed (Rani et al., 2017; Fiorentino et al., 2019; Pesce et al., 2019).

While the V3-V4 hypervariable regions of 16S rRNA are frequently used for human microbiome profiling, sequencing only two fragments of the 16S rRNA gene does not allow to narrow down the classified the bacteria to the species level due to the homology between sequences of phylogenetically related bacteria and/or issues related to bacterial nomenclature and taxonomy (Vetrovsky and Baldrian, 2013; Bukin et al., 2019; Winand et al., 2020). Many investigators have found resolution problems at the genus and/or species level with 16S rRNA gene sequencing data (Janda and Abbott, 2007; Liu et al., 2020), especially for the family Enterobacteriaceae (in particular, *Enterobacter* and *Pantoea*), *Acinetobacter baumannii*—*A. calcoaceticus complex, Achromobacter, Stenotrophomonas,* and *Actinomyces* (Chang et al., 2005; Janda and Abbott, 2007). In addition, 16S rRNA gene sequencing cannot distinguish between recently diverged species (Peker et al., 2019). The difference between the closest and next closest match to the unknown strain is < 0.5% divergence (>99.5% similarity) (Petti, 2007; Paul et al., 2019). In these circumstances, such small differences cannot justify choosing the closest match as a definitive identification. Since classification to the species level based on V3-V4 hypervariable regions of 16S rRNA is debatable and usually not achievable (Hiergeist et al., 2023), we performed data analysis at the bacterial genus level. Also, due to the inability to quantify microorganisms in amplicon sequencing, only qualitative assessment of microorganisms identified by this method was performed. Thus, in this study, data analysis at the level of bacterial genus has been performed. Also, due to the inability to quantify microorganisms in amplicon sequencing, only qualitative analyzes of microorganisms identified by this method were performed.

Summarizing, our study provides evidence of the diversity of bacterial composition in urine samples in patients undergoing dialysis and patients after kidney transplantation compared to unaffected individuals. The applied amplicon sequencing allowed a better characterization of the composition of urobiome than classical microbiology methods. *Leptotrichia, Finegoldia,* and *Corynebacterium* were revealed as discriminative bacteria in patients undergoing dialysis and after kidney transplantation with UTI. This latest finding may form the basis of a future diagnostic process involving these bacteria as diagnostic biomarkers or in future treatment strategies for the diseases discussed here.

## Data availability statement

The datasets presented in this study can be found in online repositories. The names of the repository/repositories and accession number(s) can be found at: BioProject: PRJNA949101.

## Ethics statement

The study protocol was approved by the Bioethics Committee at Poznan University of Medical Sciences (nr 942/14 (04.12.2014), nr 191/15 (05.02.2015) and 1170/19 (05.12.2019)). The possible consequences of the study were explained, and informed consent was obtained from all participants, according to the Declaration of Helsinki. The patients/participants provided their written informed consent to participate in this study.

## Author contributions

MJ and MG designed the experiment. MJ and PP collected the urine samples and prepared bacterial genetic material to 16S rRNA sequencing. KJ and MR performed the amplicon sequencing, supervised by RP. MK and KP performed medical examination and enrolled patients in this study. MJ conducted experiments using classical microbiology. DN-M performed the statistical analysis. MJ, JK, and MG participated in the data analysis. MJ and JK wrote the manuscript. MG revised the manuscript. All authors contributed to the article and approved the submitted version.

## Funding

This research was funded by the National Science Centre in Poland, grant no. 2015/19/N/NZ6/01780 (to MJ).

## Conflict of interest

The authors declare that the research was conducted in the absence of any commercial or financial relationships that could be construed as a potential conflict of interest.

## Publisher’s note

All claims expressed in this article are solely those of the authors and do not necessarily represent those of their affiliated organizations, or those of the publisher, the editors and the reviewers. Any product that may be evaluated in this article, or claim that may be made by its manufacturer, is not guaranteed or endorsed by the publisher.
