# Supplementary material for: Differences in the composition of the bacterial element of the urinary tract microbiome in patients undergoing dialysis and patients after kidney transplantation
Source: Front Microbiol. 2023 Jun 7;14:1187625. doi: 10.3389/fmicb.2023.1187625 (PMC10282556; doi:10.3389/fmicb.2023.1187625)
Supplement: Supplementary file 2 [file Table_2.docx]

Supp. Table 2. Bacteria identified in urine samples of patients after kidney transplantation

| **Patient ID** | **<10^5 cfu/ml** | **<10^3 cfu/ml** |
| --- | --- | --- |
| 1 | - | - |
| 2 | - | *-* |
| 3 | - | *Dermacoccus* spp.*, Micrococcus luteus* |
| 4 | - | *Staphylococcus epidermidis* |
| 5 | - | - |
| 6 | - | *Staphylococcus haemolyticus* |
| 7 | - | *Escherichia coli, Enterococcus faecalis, Staphylococcus epidermidis* |
| 8 | - | - |
| 9 | *Kocuria kristinae* | - |
| 10 | - | - |
| 11 | *Kocuria kristinae* | *Enterococcus faecalis* |
| 12 | *Escherichia coli* | *Staphylococcus hominis* |
| 13 | - | *Gardnerella vaginalis* |
| 14 | - | *Escherichia coli* |
| 15 | - | *Staphylococcus hominis, Enterococcus faecalis* |
| 16 | *Morganella morganii* spp *morganii* | *-* |
| 17 | - | *Enterococcus faecalis* |
| 18 | - | *Granulicatella adiacens* |
| 19 | - | - |
| 20 | *Morganella morganii, Klebsiella oxytoca* | *-* |
| 21 | - | *α hemolytic Streptococcus* spp. |
| 22 | - | *-* |
| 23 | - | - |
| 24 | - | - |
| 25 | *Escherichia coli* | *Kocuria kristinae, Staphylococcus haemolyticus* |
| 26 | *Escherichia coli* | - |
| 27 | - | - |
| 28 | - | *Kocuria kristinae* |
| 29 | - | *Staphylococcus aureus* |
| 30 | - | *Dermacoccus* spp. |
| 31 | *Escherichia coli* | *Citrobacter* spp. |
| 32 | - | - |
| 33 | - | *α hemolytic Streptococcus* spp. |
| 3 | - | *Staphylococcus aquorum* |
| 35 | *Escherichia coli* | *-* |
| 36 | *Escherichia coli* | *-* |
| 37 | *Escherichia coli* | *Enterococcus faecalis* |
| 38 | *Escherichia coli* | *-* |
| 39 | *Escherichia coli* | *-* |
| 40 | - | *Staphylococcus epidermidis* |
| 41 | - | - |
| 42 | - | *Staphylococcus epidermidis, Staphylococcus hominis, Streptococcus agalactiae, Enterococcus faecalis* |
| 43 | - | - |
| 44 | *Enterobacter cloacae* complex | - |
| 45 | - | - |
| 46 | - | - |
| 47 | - | *Enterococcus faecalis* |
| 48 | - | - |
| 49 | - | - |
| 50 | *Escherichia coli* | *Staphylococcus haemolyticus, Streptococcus agalactiae* |

Abbreviations: CNS, *Coagulase negative Staphylococcus*
