# Supplementary material for: Differences in the composition of the bacterial element of the urinary tract microbiome in patients undergoing dialysis and patients after kidney transplantation
Source: Front Microbiol. 2023 Jun 7;14:1187625. doi: 10.3389/fmicb.2023.1187625 (PMC10282556; doi:10.3389/fmicb.2023.1187625)
Supplement: Supplementary file 3 [file Table_3.docx]

Supp. Table 3. Bacteria identified in urine samples of control individuals

| **Patient** | **<10^5 cfu/ml** | **<10^3 cfu/ml** |
| --- | --- | --- |
| 1 | - | *Staphylococus epidermidis, CNS* |
| 2 | - | *-* |
| 3 | - | *Enterococcus* spp., *Escherichia coli* |
| 4 | - | *Enterococcus* spp., *CNS* |
| 5 | - | *-* |
| 6 | - | *Staphylococcus aureus, CNS* |
| 7 | - | *CNS, Escherichia coli* |
| 8 | - | *-* |
| 9 | - | *α hemolytic Streptococcus* spp. |
| 10 | - | *Enterococcus* spp., *CNS* |
| 11 | - | *-* |
| 12 | - | *-* |
| 13 | - | *-* |
| 14 | - | *α hemolytic Streptococcus* spp. |
| 15 | - | *-* |
| 16 | - | *-* |
| 17 | - | *Enterococcus* spp.,*CNS* |
| 18 | - | *α hemolytic Streptococcus* spp. |
| 19 | - | *CNS* |
| 20 | - | *CNS, Escherichia coli, Enterococcus* spp. |
| 21 | - | *CNS* |
| 22 | - | *-* |
| 23 | - | *Enterococcus* spp., *CNS , Escherichia coli* |
| 24 | - | *-* |
| 25 | - | *CNS ,Escherichia coli* |
| 26 | - | *-* |
| 27 | - | *-* |
| 28 | - | *CNS* |
| 29 | - | *Enterococcus* spp. |
| 30 | - | *CNS* |
| 31 | - | *-* |
| 32 | - | *Escherichia coli* |
| 33 | - | *-* |
| 34 | - | *CNS* |
| 35 | - | *CNS* |
| 36 | - | *CNS* |
| 37 | - | *Enterococcus* spp. |
| 38 | - | *CNS* |
| 39 | - | *Enterococcus* spp. |
| 40 | - | *-* |
| 41 | - | *α hemolytic Streptococcus* spp. |
| 42 | - | *Enterococcus* spp., *α hemolytic Streptococcus* spp. |
| 43 | - | *α hemolytic Streptococcus* spp. |
| 44 | - | *CNS* |
| 45 | - | *α hemolytic Streptococcus* spp. |
| 46 | - | *-* |
| 47 | - | *α hemolytic Streptococcus* spp. |
| 48 | - | *Enterococcus* spp. |
| 49 | - | *α hemolytic Streptococcus* spp. |
| 50 | - | *α hemolytic Streptococcus* spp. |

Abbreviations: CNS, *Coagulase negative Staphylococcus*
